# A simple method to separate base population and segregation effects in genomic relationship matrices

**DOI:** 10.1186/s12711-015-0130-8

**Published:** 2015-06-23

**Authors:** Laura Plieschke, Christian Edel, Eduardo CG Pimentel, Reiner Emmerling, Jörn Bennewitz, Kay-Uwe Götz

**Affiliations:** Bavarian State Research Center for Agriculture, Institute of Animal Breeding, Prof.-Dürrwaechter-Platz 1, 85586 Poing-Grub, Germany; Institute of Animal Husbandry and Breeding, University Hohenheim, Garbenstraße 17, 70599 Stuttgart, Germany

## Abstract

**Background:**

Genomic selection and estimation of genomic breeding values (GBV) are widely used in cattle and plant breeding. Several studies have attempted to detect population subdivision by investigating the structure of the genomic relationship matrix **G**. However, the question of how these effects influence GBV estimation using genomic best linear unbiased prediction (GBLUP) has received little attention.

**Methods:**

We propose a simple method to decompose **G** into two independent covariance matrices, one describing the covariance that results from systematic differences in allele frequencies between groups at the pedigree base (**G**_A_^*^) and the other describing genomic relationships (**G**_S_) corrected for these differences. Using this decomposition and F_st_ statistics, we examined whether observed genetic distances between genotyped subgroups within populations resulted from the heterogeneous genetic structure present at the base of the pedigree and/or from breed divergence. Using this decomposition, we tested three models in a forward prediction validation scenario on six traits using Brown Swiss and dual-purpose Fleckvieh cattle data. Model 0 (M0) used both components and is equivalent to the model using the standard **G**-matrix. Model 1 (M1) used **G**_S_ only and model 2 (M2), an extension of M1, included a fixed genetic group effect. Moreover, we analyzed the matrix of contributions of each base group (**Q**) and estimated the effects and prediction errors of each base group using M0 and M1.

**Results:**

The proposed decomposition of **G** helped to examine the relative importance of the effects of base groups and segregation in a given population. We found significant differences between the effects of base groups for each breed. In forward prediction, differences between models in terms of validation reliability of estimated direct genomic values were small but predictive power was consistently lowest for M1. The relative advantage of M0 or M2 in prediction depended on breed, trait and genetic composition of the validation group. Our approach presents a general analogy with the use of genetic groups in conventional animal models and provides proof that standard GBLUP using **G** yields solutions equivalent to M0, where base groups are considered as correlated random effects within the additive genetic variance assigned to the genetic base.

## Background

Genomic selection [1] and estimation of genomic breeding values (GBV) are currently used for many cattle populations. Genomic best linear unbiased prediction (GBLUP) using relationships estimated based on SNPs (single nucleotide polymorphisms) has been established as one of the most prominent methods for practical applications [2]. The question of how and to what extent population subdivision affects the genomic relationship matrix and genomic predictions was not addressed until applications of GBLUP across breeds or in admixed or crossbred populations were proposed e.g. [3–5]. However, several authors have shown that genomic relationship matrices can be used to detect population subdivision and to calculate measures of genetic distances (e.g. F_st_) [6, 7].

Conventional methods to estimate breeding values consider that animals with unknown parents belong to an arbitrarily defined base population. Members of this base population are assumed to come from a single population with a mean breeding value of 0 and variance *σ*_*a*_^2^. Since this is rarely true in practical applications, many conventional methods to estimate breeding values include genetic groups or phantom parents [8–10] in the model. A more elaborated approach in the context of multi-breed evaluations was proposed by García-Cortés and Toro [11], who partitioned the elements of the covariance matrix of the additive values into a breed-source term and a segregation term.

In spite of the large number of studies that deal with the use of genetic groups in conventional models, only a few have investigated this issue within the framework of genomic models. Makgahlela et al. [12–14] tested models that accounted for breed effects and compared allele frequencies in subgroups of Nordic Red cattle. They showed that a model that included a fixed breed effect [12, 13] increased the reliability of direct genomic values (DGV) by 2 to 3 % [13] for an admixed Nordic Red population. In a follow-up investigation, they found that using breed- or subpopulation-specific allele frequencies to calculate the genomic relationship matrix (**G**) did not result in higher validation reliabilities, although accounting for specific allele frequencies in the calculation of **G** changed the estimated GBV of some individuals considerably [14]. Tsuruta et al. [15] proposed an approach to assign unknown parent groups in one-step GBLUP for US Holstein cattle data. Their approach can be described as an application of the model that fits standard fixed genetic groups within the context of one-step GBLUP. The question of whether and how population subdivision influences the **G-**matrix was not addressed.

A simulation study by Vitezica et al. [16] compared five BLUP methods and investigated the effect of selection and genome-wide evaluation methods (one-step and multi-step) on bias and accuracy of genomic predictions. They examined the problem of unequal genetic levels between genotyped and non-genotyped animals in the one-step GBLUP procedure, where the genomic relationship matrix **G** and the pedigree-based relationship matrix **A** are combined. They proposed a correction of **G** and concluded that one-step estimation with a corrected **G** results in unbiased estimates of GBV, which have a similar inflation rate and a higher accuracy than estimates obtained with other methods. Christensen [17] presented an alternative approach for one-step models. For admixed populations, he suggested that the pedigree-based relationship matrix should be adjusted by assuming a parametric structure for the relationships between animals in the base population and estimating those parameters. He argued that this approach would be easier to extend and simpler than developing an appropriate method of adjusting the matrix of genomic relationships of genotyped animals across breeds.

The effects of population subdivision on the structure of the genomic relationship matrix **G** have also been investigated in contexts other than when it is used to estimate GBV. There are numerous studies on the calculation of F_st_ statistics [6, 18] and principal component analysis (PCA), e.g. [19, 20], and corresponding extensions to the **G-**matrix [16]. These studies show that it is possible to detect population subdivision with **G** in the same manner as with **A**. This means that **G** includes information about population subdivision and that, in some cases, this information includes the genetic distance between potentially discriminable groups in the base population that is defined by the pedigree. Since base animals are rarely genotyped, these distances cannot be estimated directly. A simple and straightforward method to estimate allele frequencies in the base population was proposed by Gengler et al. [21] and is based on a mixed model approach. In this paper, we estimate allele frequencies in the base of different subpopulations that are present in our datasets and propose a method to separate the genomic relationship matrix (**G**) into two independent components: a base group (**G**_A_^*^) component and a segregation (**G**_S_) component. Furthermore, we demonstrate that this decomposition leads to basically identical results as ordinary GBLUP. Finally, we examine models that either ignore the effects of base groups or that consider base groups as fixed effects.

## Methods

### Material

In total, 7965 genotyped Fleckvieh (FV) and 4257 genotyped Brown Swiss (BS) and 143 genotyped Original Braunvieh (OB) bulls were available for this study. BS and OB data were combined (hereafter called BS/OB, *n* = 4400) into a single dataset because these two subpopulations actually originated from a single breed. The term Brown Swiss is used to denote the modern Braunvieh, which resulted from an exchange of genetic material between Europe and North America. An OB animal is genetically characterized as a descendant of the old European Braunvieh population, with no or only minor genetic contributions from the reimported US Brown Swiss population. This labelling of OB animals within the European Braunvieh population is not necessarily applied in a uniform manner and small differences in the definition can occur between countries.

All animals were genotyped with the Illumina BovineSNP50 BeadChip (Illumina, San Diego, CA). After removing SNPs with low call rates (<90 %), minor allele frequencies less than 2 %, or with a deviation from Hardy-Weinberg equilibrium with *P* < 10^−5^, 37 718 and 41 254 SNPs were retained for the BS/OB and FV datasets, respectively. Available pedigrees for genotyped animals included 7802 and 16 357 records for the BS/OB and FV breeds, respectively. BS/OB base animals were assigned to nine groups (Table 1) according to origin and date of birth. Since the genetic distances between German, Austrian, Italian and Swiss BS base animals born before 1960 were small (results not shown), they were combined into one base group called EU_b_. Base FV animals were assigned to 11 groups with nine groups assigned according to origin and date of birth and two groups assigned to the Red Holstein breed (Table 2).

**Table 1:** Number of animals per defined base group for the BS/OB population

	EU_b_	DE_b_	AT_b_	CH_b_	IT_b_	US_b1_	US_b2_	OB_b1_	OB_b2_
Year	≤1960	>1960	>1960	>1960	>1960	≤1955	>1955	≤1960	>1960
Number	2093	1482	743	1281	413	489	445	458	398

*BS* = Brown Swiss and *OB* = Original Braunvieh, assignment was done by country and year of birth with the exception of the OB base groups, which were considered across countries: *EU*_*b*_ = European base group (born before 1960), *DE*_*b*_ = German base group (born after 1960), *AT*_*b*_ = Austrian base group (born after 1960), *CH*_*b*_ = Swiss base group (born after 1960), *IT*_*b*_ = Italian base group (born after 1960), *US*_*b1*_ = American base group (born before 1955), *US*_*b2*_ = American base group (born after 1955), *OB*_*b1*_ = Original Braunvieh base group (born before 1960), *OB*_*b2*_ = Original Braunvieh base group (born after 1960)

**Table 2:** Number of animals per defined base group for FV

	DE_b1_	DE_b2_	DE_b3_	DE_b4_	HOL_b1_	HOL_b2_	AT_b_	CZ_b_	CH_b_	FR_b_	Div_b_
Year	<1960	≥1960 < 1970	≥1970 < 1980	≥1980	<1960	≥1960	All	All	All	All	All
Number	1368	6055	1661	773	528	427	3452	977	183	228	705

*FV* = Fleckvieh; assignment was done by country and year of birth with the exception of the Red Holstein and the diverse base groups, which were considered across countries: *DE*_*b1*_ = German base group (born before 1960), *DE*_*b2*_ = German base group (born between 1960 and 1970), *DE*_*b3*_ = German base group (born between 1970 and 1980), *DE*_*b4*_ = German base group (born after 1980), *HOL*_*b1*_ = Red Holstein base group (born before 1960), *HOL*_*b2*_ = Red Holstein base group (born after 1960), *AT*_*b*_ = Austrian base group, *CZ*_*b*_ = Czech base group, *CH*_*b*_ = Swiss base group, *FR*_*b*_ = French base group, *DIV*_*b*_ = base groups with animals with other countries of origin

We estimated DGV for three milk traits and three conformation traits from a dataset that was reduced for the last four years of phenotypic data (referred to as the reduced dataset). Daughter yield deviations (DYD) from the German-Austrian system [22] were used for FV bulls and deregressed MACE (multi-trait across country evaluations) proofs from Interbull [23] for BS/OB bulls. Deregression was done using the method proposed by Garrick et al. [24]. Group effects were not accounted for in the deregression. Traits analyzed were milk yield (MY), protein yield (PY), fat yield (FY), stature (STA), feet and legs (FL) and udder conformation (UD). These traits were *a priori* assumed to have a large genetic trend and/or to show considerable differences between base groups. DGV estimated from the reduced dataset were then compared to DYD and deregressed proofs from the corresponding April 2014 evaluations (current dataset) according to the guidelines of the Interbull GEBV test [25, 26]. In short, the validation group included bulls with no information on the offspring’s performances in the reduced dataset but corresponding information in the current dataset. Current information was assumed to be sufficient for the test when the effective daughter contribution (EDC) [27] based on offspring performances was equal to at least 20. The remaining bulls from 2010 with an EDC of at least 1 were included into the training set (*Calib*).

Technically, we tested DGV by a weighted regression of current DYD or deregressed proofs of the animals in the validation group on their DGV estimated from the reduced set. The resulting test statistics are the intercept and slope (b) of this regression as measures of bias and the coefficient of determination (R^2^) of this regression as a measure of the reliability of the DGV. The R^2^ values were corrected for the uncertainty in DYD, as proposed by [28], i.e. they were divided by the average reliability of the DYD of validation bulls.

For presentation of results, we divided the animals of the validation group into different sub-groups. FV validation animals were assigned to two groups: animals from Germany-Austria (*DEA*) and *others*. BS validation animals were also divided into *DEA* and *others*, and OB validation animals were assigned to a third validation group (*OB*). Numbers of animals included in each validation group are in Table 3. The assignments of validation animals to origins used in this investigation for the purpose of illustration were mainly based on ISO country codes [29] and do not necessarily correspond to assignments based on analyses of genetic contributions from base groups.

**Table 3:** Number of animals per validation group for the BS/OB and FV populations and the seven traits considered

		Training set	Validation set		
			*DEA*	*others*	*OB*
BS/OB	MY	3262	416	346	8
	PY	3262	416	346	8
	FY	3262	416	346	8
	STA	3535	464	350	51
	FL	3551	461	345	43
	UD	3550	458	349	43
			*DEA*	*others*	**-**
FV	MY	5276	2589	97	
	PY	5276	2581	97	-
	FY	5276	2581	97	
	STA	5956	2264	139	-
	FL	5956	2272	139	
	UD	5956	2272	139	-

*BS* = Brown Swiss, *OB* = Original Braunvieh and *FV* = Fleckvieh, *MY* = milk yield, *PY* = protein yield, *FY* = fat yield, *STA* = stature, *FL* = feet and legs, *UD* = udder conformation

Validation sets: *DEA* = German and Austrian validation animals; *others* = validation animals with other countries of origin; *OB* = Original Braunvieh validation animals

### Decomposition of G

Assume a common scenario in genomic prediction with *n* animals genotyped for *m* biallelic SNPs. Information on genotypes is collected in an *n* x *m* matrix **C**, using numerical coding that denotes the number of copies of the arbitrarily defined reference allele (0, 1, 2). Let **p**_T_ be the vector of estimated allele frequencies at the *m* SNPs, which for each SNP *j* were derived from genotyped animals.

1 $$ {\widehat{\mathrm{p}}}_{\mathrm{j}}=\frac{{\displaystyle {\sum}_{\mathrm{i}=1}^{\mathrm{n}}{\mathrm{C}}_{\mathrm{i}\mathrm{j}}}}{2\mathrm{n}} $$

A genomic relationship matrix **G**_T_ can be calculated and used in GBLUP using these “current” allele frequencies as:

2 $$ {\mathbf{G}}_{\mathrm{T}} = \frac{\mathbf{MM}\hbox{'}}{{\displaystyle {\sum}_{\mathrm{j}=1}^{\mathrm{m}}2{\widehat{\mathrm{p}}}_{\mathrm{j}}\left({1\hbox{-} \widehat{\mathrm{p}}}_{\mathrm{j}}\right)}}, $$

where **M** is an *n* x *m* matrix of recoded genotypes, for which each row (= animal) *i* of the matrix of numerically coded genotypes **C** is manipulated in the following manner [30]:

3 $$ {\mathbf{M}}_{\mathrm{i}} = {\mathbf{C}}_{\mathrm{i}}\ \hbox{-} 1\hbox{-}\ 2\left({\mathbf{p}}_{\mathrm{T}}\hbox{-}\ 0.5\right). $$

Conceptually, this manipulation is equivalent to column-wise centering of **C** if current allele frequencies are used and if each marker is in Hardy-Weinberg equilibrium in the genotyped population.

Assume a subdivision of the genotyped population into *g* groups that systematically differ in allele frequencies, as indicated for example by sufficiently high F_st_ values [31, 32]. Define a *g* x *m* matrix **P** of group-specific allele frequencies that are derived by applying Equation (1) within each group. Using these group-specific allele frequencies, the vector of genotypes for each animal can then be centered by applying Equation (3) using the allele frequencies of the group that it is assigned to. Thus, for animal *i* assigned to group *k* with group-specific allele frequencies **p**_k_, the corresponding row in **C** is manipulated as:

$$ {\mathbf{M}}_{\mathrm{i}}^{*} = {\mathbf{C}}_{\mathrm{i}}\hbox{-}\ 1\hbox{-} 2\left({\mathbf{p}}_{\mathrm{k}}\hbox{-} 0.5\right). $$

A **G**-matrix corrected for specific allele frequencies for different groups can then be calculated as:

4 $$ {\mathbf{G}}_{\mathrm{S}} = \frac{{\mathbf{M}}^{*}{\mathbf{M}}^{*\mathbf{\hbox{'}}}}{{\displaystyle {\sum}_{\mathrm{j}=1}^{\mathrm{m}}2{\widehat{\mathrm{p}}}_{\mathrm{j}}\left({1\hbox{-} \widehat{\mathrm{p}}}_{\mathrm{j}}\right)}}, $$

with the same denominator as in Equation (2), which is equivalent to expressing this part of the covariance relative to the overall covariance. The discarded component of the original covariance structure, which is caused by differences between group allele frequencies and overall frequencies, can be summarized in a matrix **G**_A_. Treating 2**P** as a matrix of average “genotypes” of groups, a matrix $\tilde{\mathrm{M}}$ is calculated by manipulating each group’s row g as follows:

$$ {\tilde{\mathbf{M}}}_{\mathrm{g}} = {\left(2\mathbf{P}\right)}_{\mathrm{g}}\hbox{-}\ 1\hbox{-}\ 2\left({\mathbf{p}}_{\mathrm{T}}\hbox{-}\ 0.5\right). $$

Finally, **G**_A_ is calculated as $\tilde{\mathbf{M}}\tilde{\mathbf{M}}\hbox{'}$ divided by the same denominator as in Equations (2) and (4). The *g* x *g* matrix **G**_A_ can be treated and analyzed in the same manner as the standard **G-**matrix. It can be expanded to give an *n* x *n* matrix **G**_A_^*^ based on:

$$ {\mathbf{G}}_{\mathrm{A}}^{*}=\mathbf{Q}{\mathbf{G}}_{\mathrm{A}}\mathbf{Q}\hbox{'}, $$

where **Q** is the matrix of genetic contributions of each base group to each animal, which can be calculated as:

$$ \mathbf{Q} = \mathbf{T}{\mathbf{Q}}^{*}, $$

where **T** is a lower triangular matrix that results from decomposing **A** into **TDT’**, as described in [33], and Q^*^ is an *n x* g design matrix that assigns genotyped animals to groups. Despite this increase in dimensions, **G**_A_^*^ still has rank (*g* – 1). Also, note that:

5 $$ {\mathbf{G}}_{\mathrm{T}} = {\mathbf{G}}_{\mathrm{S}} + {\mathbf{G}}_{\mathrm{A}}^{*}. $$

Although this decomposition is straightforward, its dependency on the current allele frequencies and the grouping of current animals causes some problems due to ambiguous genetic composition and might not be feasible under practical conditions since new genotypes have to be successively integrated into the system. To circumvent this problem, we propose to replace the current allele frequencies with estimates of base allele frequencies using the estimation procedure developed by Gengler et al. [21]. Using a pedigree that relates genotyped animals to a set of arbitrarily defined but usually ungenotyped base animals and calculating the conventional relationship matrix **A**, the vector of overall base allele frequencies is calculated as a generalized least squares mean by solving the following equation for each marker *j* (column of **C**):

6 $$ {\mathrm{p}}_{\mathrm{j}}^{*}=0.5\ \left[{\left({\mathbf{1}}^{\hbox{'}}{\mathbf{A}}^{\hbox{-} 1}\mathbf{1}\right)}^{\hbox{-} 1}\ {\mathbf{1}}^{\hbox{'}}{\mathbf{A}}^{\hbox{-} 1}\ {\mathbf{c}}_{\mathrm{j}}\right]. $$

Similar to conventional estimation of GBV, base animals can be grouped according to known or assumed population subdivisions and/or generations, when additional differentiation due to considerable genetic trend has to be taken into account. To estimate base group-specific allele frequencies, matrix **1** in Equation (6) is replaced by matrix **Q**. Matrices **G**_**T**_, **G**_S_ and **G**_A_^*^ can then be calculated as described above, using estimates for global and group-specific base allele frequencies and again **G**_T_ = **G**_S_ + **G**_A_^*^, as described above.

### Models

In order to study the influence of different definitions of base group on the quality of prediction, we examined several models. The general model is a standard mixed animal model with:

$$ \mathbf{y}=\mathbf{X}\mathbf{b}+\mathbf{Z}\mathbf{u}+\mathbf{e}, $$

where **y** is a vector of DYD or deregressed proofs of genotyped animals, **b** is the vector of fixed effects, **u** is the vector of random animal effects, incidence matrices **X** and **Z** relate observations to levels of **b** and **u**, respectively, and **e** is the residual effect. Furthermore, it is assumed that **y** ~ N(**Xb**, **V**_**yy**_), **e** ~ N(**0**,**V**_**e**_) and **u** ~ N(**0**,**V**_**uu**_), with **V**_**yy**_ = **V**_**uu**_ + **V**_**e**_, **V**_**e**_ is diag(**1**/**w**)*σ^2^_e_, where **w** is a vector of weights. The models to be compared are defined in the following.

- Standard model (model 0, M0): X = 1 and V_uu_ = G_T_ × *σ*_*u*_^2^.
- Model 1 (M1): X = 1 and V_uu_ = G_S_ × *σ*_*u*_^2^.
- Model 2 (M2): X = [1 | Q] and V_uu_ = G_S_ × *σ*_*u*_^2^.

Note that M2 is equivalent to a model that fits standard fixed group effects [34]. Although genomic relationships corrected for unequal base allele frequencies (**G**_S_) are used in M2, it can be shown by least-squares theory that the solutions are identical to a model that uses **G**_T_, if the same matrix **Q** is used to estimate the base allele frequencies and to model the fixed group effects (see Appendix 1). Finally, it can be shown that using the standard genomic relationship matrix **G**_**T**_ in standard GBLUP (standard model, M0) in the presence of base groups that differ in allele frequencies gives solutions equivalent to the use of a more specific model with genetic groups as random effects and equal variances for the base group and the segregation effects (see Appendix 2), as in the following representation:

$$ \left[\begin{array}{ccc}\hfill \mathbf{X}\mathbf{\hbox{'}}\mathbf{X}\hfill & \hfill \mathbf{X}\mathbf{\hbox{'}}\mathbf{Z}\hfill & \hfill \mathbf{X}\mathbf{\hbox{'}}\mathbf{Q}\ \hfill \\ {}\hfill \mathbf{Z}\mathbf{\hbox{'}}\mathbf{X}\hfill & \hfill \mathbf{Z}\mathbf{\hbox{'}}\mathbf{Z} + {\mathbf{G}}_{\mathrm{S}}{}^{\hbox{-} 1}\hfill & \hfill \mathbf{Z}\mathbf{\hbox{'}}\mathbf{Q}\hfill \\ {}\hfill \mathbf{Q}\mathbf{\hbox{'}}\mathbf{X}\hfill & \hfill \mathbf{Q}\mathbf{\hbox{'}}\mathbf{Z}\hfill & \hfill \mathbf{Q}\mathbf{\hbox{'}}\mathbf{Q} + {\mathbf{G}}_{\mathrm{A}}{}^{\hbox{-} 1}\uplambda \hfill \end{array}\right]\left[\begin{array}{c}\hfill \widehat{\mathbf{b}}\hfill \\ {}\hfill \widehat{\mathbf{u}}\hfill \\ {}\hfill \widehat{\mathbf{g}}\hfill \end{array}\right]=\left[\begin{array}{c}\hfill \mathbf{X}\mathbf{\hbox{'}}\mathbf{y}\hfill \\ {}\hfill \mathbf{Z}\mathbf{\hbox{'}}\mathbf{y}\hfill \\ {}\hfill \mathbf{Q}\mathbf{\hbox{'}}\mathbf{y}\hfill \end{array}\right], $$

where λ = *σ*_*u*_^2^/*σ*_*e*_^2^ and the final estimate for the breeding value is  = **Qĝ** + **û**. We calculated solutions for the standard model using this more specific model, which, in addition, allowed us to derive estimates for group effects and their prediction errors.

Models were tested in forward prediction by means of the test described in the sub-section Material. To better understand the factors that influence the predictive ability of a specific model for different validation datasets, we analyzed the matrix of base group contributions (**Q**) and derived base group estimates, as well as their prediction errors, using M0 and M2. Differences between group effect estimates were calculated and tested by formulating linear hypotheses.

### Distance measures

We calculated F_st_ statistics to illustrate the effects of the proposed decomposition of **G**. F_st_ is a standard measure of genetic distance and can be calculated either by pairwise analysis of differences in allele frequencies between known or assumed subpopulations or breeds [18], or by direct calculation from relationship matrices [6] as:

$$ {\mathrm{F}}_{\mathrm{st}} = \frac{\tilde{\mathrm{f}}\hbox{-} \overline{\mathrm{f}}}{1\hbox{-} \overline{\mathrm{f}}}, $$

where $\tilde{\mathrm{f}}$ is the mean coancestry over all subpopulations and $\overline{\mathrm{f}}$ is the average coancestry within a given subpopulation. The term $1\hbox{-} \tilde{\mathrm{f}}$ is the average diversity (heterozygosity) and depends on the coancestry within the given subpopulation. F_st_ values are primarily used as a tool to visualize substructures within groups of animals [6, 10, 35]. An F_st_ value of 0.05 can be interpreted as a strong indication of a relevant subdivision [31, 32].

## Results

### F_st_ statistics

**Fig. 1:**
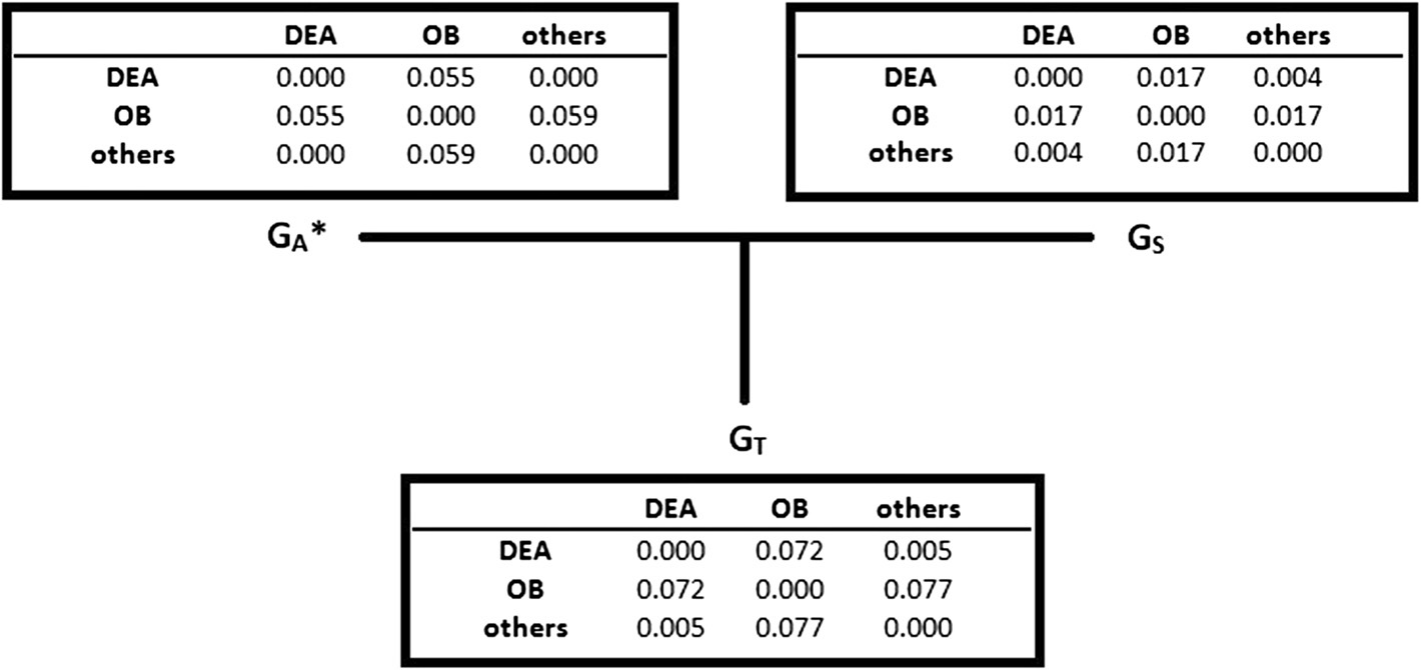
F_st_ values of the base group term (**G**_A_^*^), the segregation term (**G**_**S**_), and total **G** (**G**_**T**_) for the 4400 BS/OB animals. BS = Brown Swiss and OB = Original Braunvieh

To illustrate the effects of the decomposition of the **G**-matrix, we calculated F_st_ values for both components (**G**_S_ and **G**_A_^*^) and for the total **G**-matrix for the 4400 BS/OB animals. Results of the F_st_ statistics are in Fig. 1. Comparison of distances calculated from **G**_A_^*^ and **G**_**S**_ shows that population differences were primarily caused by genetic distances in the base population. A substantial genetic distance existed only between the OB group and the two other groups. This distance was present in both **G**_A_^*^ and **G**_**S**_**,** but was considerably greater in **G**_A_^*^. Interestingly, the distances in **G**_A_^*^ and **G**_**S**_ acted additively and their sum resulted in the distances calculated from **G**_**T**_.

### Forward prediction

Results of the forward prediction in terms of the coefficient of determination (R^2^), the intercept (a), the slope (b) and corresponding standard errors are in Tables 4 and 5. For both breeds and across all traits, differences between models were small, but M1 consistently resulted in a lower R^2^.

**Table 4:** Results for the coefficient of determination (R^2^) from the forward prediction for the BS/OB and FV populations for different models

BS/OB	Trait	M0 (**G**_A_^*^ and **G**_**S**_)	M1 (**G**_**S**_)	M2 (**G**_**S**_ + fixed effects)
R^2^	MY	0.416	0.386	0.421
	PY	0.409	0.370	0.417
	FY	0.388	0.349	0.395
	STA	0.499	0.382	0.505
	FL	0.234	0.216	0.220
	UD	0.416	0.394	0.410
FV				
R^2^	MY	0.580	0.530	0.557
	PY	0.512	0.463	0.491
	FY	0.548	0.490	0.521
	STA	0.526	0.515	0.516
	FL	0.438	0.425	0.415
	UD	0.406	0.404	0.405

*BS* = Brown Swiss, *OB* = Original Braunvieh, and *FV* = Fleckvieh, *MY* = milk yield, *PY* = protein yield, *FY* = fat yield, *STA* = stature, *FL* = feet and legs, *UD* = udder conformation

**Table 5:** Results for the intercept (a), slope (b) and its standard error (s.e.) from the forward prediction for the FV and BS/OB populations for different models

	Trait	M0 (**G**_A_^*^ and **G**_**S**_)		M1 (**G**_**S**_)		M2 (**G**_**S**_ + fixed effects)	
BS/OB		a	b (s.e.)	a	b (s.e.)	a	b (s.e.)
	MY	85.551	**0.828** (0.035)	87.672	**0.813** (0.037)	85.091	**0.820** (0.035)
	PY	3.152	**0.768** (0.033)	3.221	**0.748** (0.035)	3.129	**0.765** (0.033)
	FY	3.202	**0.762** (0.035)	3.198	**0.753** (0.037)	3.178	**0.757** (0.034)
	STA	14.934	**0.854** (0.029)	−3.706	**1.020** (0.044)	18.807	**0.817** (0.028)
	FL	1.285	**0.979** (0.061)	−4.480	**1.032** (0.068)	24.889	**0.751** (0.059)
	UD	22.008	**0.786** (0.032)	9.036	**0.904** (0.038)	30.023	**0.711** (0.030)
FV		a	b (s.e.)	a	b (s.e.)	a	b (s.e.)
	MY	62.576	**0.660** (0.019)	76.031	**0.582** (0.018)	76.031	**0.619** (0.018)
	PY	3.213	**0.664** (0.019)	3.914	**0.593** (0.019)	3.914	**0.644** (0.019)
	FY	2.640	**0.734** (0.019)	3.696	**0.650** (0.019)	3.696	**0.729** (0.020)
	STA	0.046	**0.782** (0.024)	0.076	**0.774** (0.024)	0.076	**0.786** (0.025)
	FL	−0.082	**0.900** (0.036)	−0.179	**0.878** (0.036)	−0.179	**1.021** (0.038)
	UD	−0.013	**0.713** (0.033)	−0.031	**0.708** (0.033)	−0.031	**0.736** (0.040)

*BS* = Brown Swiss, *OB* = Original Braunvieh and *FV* = Fleckvieh; values for the slope are printed in bold and values for the standard error of the slope are shown in brackets. *MY* = milk yield, *PY* = protein yield, *FY* = fat yield, *STA* = stature, *FL* = feet and legs, *UD* = udder conformation

#### Brown Swiss and Original Braunvieh breeds

For the BS/OB data, we found a minimal advantage in terms of the R^2^ for model M2 that fitted fixed groups. Exceptions were for the traits FL and UD, here the standard random model M0 showed the highest R^2^. Across traits, R^2^ for M1 was 0.028 to 0.123 lower than that of the best model. Based on results in terms of slope, it should be noted that inflation of genomic predictions was lowest for conformation traits using model M1. For milk traits, the slope was slightly higher and estimates were thus less inflated with the random model M0 than with the fixed model M2.

#### Fleckvieh breed

Differences in R^2^ between M0 and M2 ranged from 0.001 to 0.021. For all six traits, M0 resulted in a higher R^2^ than the fixed group model M2. The R^2^ achieved with M1 was always lower than that achieved with M0 and M2. Nevertheless, the difference in R^2^ between M1 and M0 was only 0.002 for the UD trait. For the other traits, the R^2^ that was achieved with M1 was between 0.011 and 0.058 lower than that with M0. Based on slope, model M0 was superior and always led to the lowest inflation of estimates for milk traits. For conformation traits, the fixed model M2 led to the lowest inflation. However, differences between models were relatively small in many cases (between 0.004 and 0.143).

### Base group effects

We estimated base group effects based on M0 and M2. Properties of matrix **Q** always lead to linear dependencies and no unique solution can be achieved. However, significant differences between group estimates can be derived and tested using linear hypotheses. Results in Tables 6 and 7 are group differences estimated with M2.

**Table 6:** Differences between base group effects estimated with the fixed model for the BS/OB population for protein yield above the diagonal and stature below the diagonal

	EU_b_	DE_b_	AT_b_	CH_b_	IT_b_	US_b1_	US_b2_	OB_b1_	OB_b2_
	≤1960	>1960	>1960	>1960	>1960	≤1955	>1955	≤1960	>1960
EU_b_	0	−64.86***	−22.52***	−13.97***	−19.36***	−26.06***	−29.90***	−14.01***	−45.54***
DE_b_	25.48***	0	42.35***	50.90***	45.50***	38.80***	34.97***	50.85***	19.32***
AT_b_	15.66***	−9.82***	0	8.55***	3.15^n.s^.	−3.55^n.s.^	−7.38 ^n.s^.	8.50*	−23.03***
CH_b_	1.21*	−24.27***	−14.45***	0	−5.40**	−12.10***	−15.93***	−0.05^n.s^.	−31.58***
IT_b_	19.63***	−5.85***	3.97***	18.42***	0	−6.70*	−10.53***	5.35*	−26.18***
US_b1_	11.23***	−14.25***	−4.43***	10.02***	−8.40***	0	−3.83^n.s.^	12.05**	−19.48***
US_b2_	23.05***	−2.43 ^n.s.^	7.39***	21.85***	3.42*	11.82***	0	15.88***	−15.65***
OB_b1_	3.56***	−21.92***	−12.11***	2.35***	−16.08***	−7.67***	−19.50***	0	−31.53***
OB_b2_	18.05***	−7.43***	2.38***	16.83***	−1.59**	6.82***	−5.01***	14.49***	0

*BS* = Brown Swiss and *OB* = Original Braunvieh; Protein yield (in kg); Stature (in cm); we calculated the differences row minus column, so negative values indicate superior horizontal groups and positive values indicate superior vertical groups. ^n.s.^ = not significant, * = (*p* < .05), ** = (*p* < .01), *** = (*p* < .001). *EU*_*b*_ = European base group (born before 1960), *DE*_*b*_ = German base group (born after 1960), *AT*_*b*_ = Austrian base group (born after 1960), *CH*_*b*_ = Swiss base group (born after 1960), *IT*_*b*_ = Italian base group (born after 1960), *US*_*b1*_ = American base group (born before 1955), *US*_*b2*_ = American base group (born after 1955), *OB*_*b1*_ = Original Braunvieh base group (born before 1960), *OB*_*b2*_ = Original Braunvieh base group (born after 1960)

**Table 7:** Differences between base group effects estimated with the fixed model for the FV population for protein yield above the diagonal and stature below the diagonal

	DE_b1_	DE_b2_	DE_b3_	DE_b4_	HOL_b1_	HOL_b2_	AT_b_	CZ_b_	CH_b_	FR_b_	Div_b_
	<1960	≥1960 < 1970	≥1970 < 1980	≥1980	<1960	≥1960	All	All	All	All	All
DE_b1_	0	−16.77***	1.06 ^n.s.^	−7.49***	−50.43***	−49.94***	18.21***	−32.21***	10.89***	−28.14***	49.76***
DE_b2_	−0.29^n.s.^	0	17.83***	9.28**	−33.66***	−33.17***	34.98***	−15.45***	27.66***	−11.37***	66.54***
DE_b3_	−1.60^n.s.^	−1.31 ^n.s.^	0	−8.55***	−51.49***	−51.00***	17.15***	−33.28***	9.82***	−29.20***	48.701***
DE_b4_	−0.24^n.s.^	0.05 ^n.s.^	1.36 ^n.s.^	0	−42.94***	−42.45***	25.70***	−24.73***	18.38***	−20.65***	57.25***
HOL_b1_	5.16***	5.45***	6.76***	5.40***	0	0.49^n.s.^	68.64***	18.21***	61.32***	22.29***	100.19***
HOL_b2_	−1.49^n.s.^	−1.20^n.s.^	0.11^n.s.^	−1.25^n.s.^	−6.65***	0	68.15***	68.14***	60.83***	21.80***	99.70***
AT_b_	−0.14 ^n.s.^	0.16^n.s.^	1.46^n.s.^	0.11^n.s.^	−5.30***	1.35^n.s.^	0	−50.43***	−7.32**	−46.35***	31.55***
CZ_b_	−3.48***	−3.19^n.s.^	−1.88^n.s.^	−3.24^n.s.^	−8.64***	−1.99^n.s.^	−3.35^n.s.^	0	43.11***	4.08^n.s.^	81.98***
CH_b_	−1.79^n.s.^	−1.50^n.s.^	−0.19^n.s.^	−1.55^n.s.^	−6.95***	−0.30^n.s.^	−1.65^n.s.^	1.69 ^n.s.^	0	−39.03***	38.88***
FR_b_	0.22^n.s.^	0.51^n.s.^	1.82^n.s.^	0.46^n.s.^	−4.95***	1.71^n.s.^	0.35^n.s.^	3.70*	2.01^n.s.^	0	77.91***
Div_b_	−3.09***	−2.80^n.s.^	−1.49^n.s.^	−2.85*	−8.25***	−1.60^n.s.^	−2.95*	0.39^n.s.^	−1.30^n.s.^	−3.31**	0

*FV* = Fleckvieh; Protein yield (in kg); Stature (in cm); we calculated the differences row minus column, so negative values indicate superior horizontal groups and positive values indicate superior vertical groups. ^n.s.^ = not significant, * = (*p* < .05), ** = (*p* < .01), *** = (*p* < .001). *DE*_*b1*_ = German base group (born before 1960); *DE*_*b2*_ = German base group (born between 1960 and 1970), *DE*_*b3*_ = German base group (born between 1970 and 1980), *DE*_*b4*_ = German base group (born after 1980), *HOL*_*b1*_ = Red Holstein base group (born before 1960), *HOL*_*b2*_ = Red Holstein base group (born after 1960), *AT*_*b*_ = Austrian base group, *CZ*_*b*_ = Czech base group, *CH*_*b*_ = Swiss base group, *FR*_*b*_ = French base group, *DIV*_*b*_ = base groups with animals with other countries of origin

#### Brown Swiss and Original Braunvieh breeds

In the BS/OB dataset, we defined nine different base groups that led to 36 possible contrasts between base groups. Differences were tested for significance using t-tests. For the PY trait, significant differences were found for the majority of group contrasts and only 5 out of 36 differences were not significant. The largest difference was between the European base group (EU_b_) and the German base group (DE_b_) (−64.86). Estimates for DE_b_ were significantly larger than estimates for all other groups. Differences between the EU_b_ group and the other groups were also large but clearly negative. The smallest difference was between the Swiss base group (CH_b_) and the older Original Braunvieh base group (OB_b1_) (−0.05). The differences between the Austrian (AT_b_) and the Italian (IT_b_) base groups were relatively small in many cases.

For the STA trait, all group differences were significant, except the difference between the German base group (DE_b_) and the younger American base group (US_b2_). The patterns of differences were quite similar as for PY, although slightly different in magnitude for STA. The largest and smallest differences were also between EU_b_ and DE_b_ (25.48) and between the Swiss base group (CH_b_) and the European base group (EU_b_) (1.21), respectively.

#### Fleckvieh breed

For the FV breed, almost all group differences were significant for PY. The largest differences were between the older Red Holstein base group (HOL_b1_) and the Austrian base group (AT_b_), between the younger Red Holstein base group (HOL_b2_) and AT_b_ and between HOL_b2_ and CZ_b_ (68.64, 68.15 and 68.14, respectively). The smallest difference was between the two Red Holstein base groups (0.49).

The situation for STA was almost the opposite. Only 16 group differences were significant, while 39 out of 55 differences were not significant. From these 16 significant differences, 10 were between the older Red Holstein base group (HOL_b1_) and all other base groups.

### Base group contributions

Analysis of the matrix of base group contributions (**Q**) revealed several general breed-specific aspects. In addition, it was possible to characterize the validation group, which can help interpretation of other results. Averages and standard deviations of base group contributions for the PY and STA traits are in Tables 8 and 9 for the two breeds.

**Table 8:** Results of the analysis of the Q-matrix for the BS/OB population

BS/OB		EU_b_	DE_b_	AT_b_	CH_b_	IT_b_	US_b1_	US_b2_	OB_b1_	OB_b2_
Year		≤1960	>1960	>1960	>1960	>1960	≤1955	>1955	≤1960	>1960
*Calib* (3262)	m	0.02	0.02	0.01	0.01	0.01	0.24	0.62	0.03	0.03
	sd	0.04	0.05	0.03	0.03	0.03	0.07	0.12	0.07	0.06
*DEA* (416)	m	0.02	0.03	0.01	0.00	0.00	0.23	0.62	0.03	0.06
	sd	0.01	0.05	0.07	0.04	0.01	0.04	0.04	0.02	0.04
*OB* (8)	m	0.25	0.00	0.01	0.05	0.00	0.00	0.00	0.54	0.16
	sd	0.25	0.00	0.02	0.09	0.00	0.00	0.00	0.19	0.15
*Others* (346)	m	0.01	0.01	0.00	0.01	0.00	0.27	0.67	0.01	0.01
	sd	0.01	0.01	0.01	0.01	0.01	0.03	0.05	0.01	0.02

*BS* = Brown Swiss and *OB* = Original Braunvieh; averages (m) and standard deviations (sd) of base group contributions are shown. *EU*_*b*_ = European base group, *DE*_*b*_ = German base group (born after 1960); *AT*_*b*_ = Austrian base group (born after 1960), *CH*_*b*_ = Swiss base group (born after 1960), *IT*_*b*_ = Italian base group (born after 1960), *US*_*b1*_ = American base group (born before 1955), *US*_*b2*_ = American base group (born after 1955), *OB*_*b1*_ = Original Braunvieh (born before 1960), *OB*_*b2*_ = Original Braunvieh (born after 1960), *Calib* = training set; Validation sets: *DEA* = German and Austrian validation animals, *others* = validation animals with other countries of origin, *OB* = Original Braunvieh validation animals

**Table 9:** Results of the analysis of the **Q**-matrix for the FV population

FV		DE_b1_	DE_b2_	DE_b3_	DE_b4_	HOL_b1_	HOL_b2_	AT_b_	CZ_b_	CH_b_	FR_b_	Div_b_
Year		<1960	≥1960 < 1970	≥1970 < 1980	≥1980	<1960	≥1960	All	All	All	All	All
*Calib* (5273)	m	0.13	0.61	0.04	0.01	0.04	0.03	0.09	0.01	0.04	0.01	0.00
	sd	0.07	0.17	0.04	0.04	0.04	0.05	0.12	0.08	0.04	0.05	0.01
*DEA* (2581)	m	0.13	0.64	0.05	0.01	0.04	0.02	0.07	0.00	0.04	0.01	0.00
	sd	0.03	0.08	0.02	0.03	0.03	0.02	0.06	0.00	0.02	0.01	0.00
*Others* (97)	m	0.07	0.36	0.02	0.00	0.09	0.08	0.05	0.25	0.04	0.03	0.02
	sd	0.03	0.14	0.02	0.01	0.05	0.07	0.04	0.13	0.03	0.06	0.02

*FV* = Fleckvieh; averages (m) and standard deviations (sd) of base group contributions are shown

*DE*_*b1*_ = German base group (born before 1960), *DE*_*b2*_ = German base group (born between 1960 and 1970), *DE*_*b3*_ = German base group (born between 1970 and 1980), *DE*_*b4*_ = German base group (born after 1980), *HOL*_*b1*_ = Red Holstein base group (born before 1960), *HOL*_*b2*_ = Red Holstein base group (born after 1960), *AT*_*b*_ = Austrian base group, *CZ*_*b*_ = Czech base group, *CH*_*b*_ = Swiss base group, *FR*_*b*_ = French base group, *DIV*_*b*_ = base groups with animals with other countries of origin, *Calib* = training set, Validation sets: *DEA* = German and Austrian validation animals, *others* = validation animals with other countries of origin

#### Brown Swiss and Original Braunvieh

In the BS population, the two American base groups (US_b1_ and US_b2_) represented between 80 % and 90 % of the overall genetic makeup of the genotyped population (Table 8). No differences in US contributions were detected between the training set (*Calib*) and the validation animals that were assigned to the *DEA* validation set and only a slight increase in US contributions was found in the *others* validation set. The small number of validation animals that was unequivocally assigned to the *OB* group showed a marked difference in this respect, with absolutely no contributions from the US base groups. Standard deviations of contributions for training animals (*Calib*) were also highest for the two US groups. Comparing standard deviations of all contributions between *Calib* and validation groups showed that the validation animals tended to have less variation, again except for the *OB* group.

#### Fleckvieh

In the FV breed, the second German base group (DE_b2_) had the largest contribution to all validation groups (Table 9). Average contributions of more than 0.60 of the second German base group to the *Calib* training set and *DEA* validation set were observed and a considerable average contribution of 0.36 to the *others* validation set. The contribution of the Czech group (CZ_b_) to the *others* validation set was relatively high (0.25).

As previously, across all base groups, we found similar average contributions to *Calib* and *DEA* and decreasing standard deviations in base group contributions when comparing *Calib* to *DEA*, which indicates an ongoing equalization of contributions.

## Discussion

In conventional methods for estimating breeding values, phantom parent groups are used in most practical applications. The reason for this is that the theoretical base population is rarely correctly represented in the available pedigree. The same is of course true for genomic evaluation models. Stratification of the population can be easily determined by F_st_ plots.

### Concept and implementation

The decomposition of the standard **G**-matrix that we propose here is primarily an analytical tool. It allows studying the following aspects in some detail: (i) whether and how differences in allele frequencies between base groups contribute to the proportion of genetic variance explained by differences between base groups; and (ii) how the effects estimated for the base groups influence the current population and their genomic predictions. Conceptually, it follows the classical approach for modeling base groups in genetic evaluations and extends it to the GBLUP case. More fundamentally, it theoretically shows that parts of the genetic variation represented by the **G**-matrix can be assigned to systematic differences in allele frequencies between base populations. This implies that standard GBLUP is equivalent to a model that fits random genetic groups, where differences in group means are modeled as part of the natural additive-genetic variance (assumed to be known in the present investigation). Recently, Makgahlela et al. [13] showed that, in the case of the largely admixed Nordic Red population, a model that fits a fixed genetic group has some advantage in terms of the reliability of DGV over the standard GBLUP model. Modeling groups as fixed might be advantageous if true differences between groups are larger than what can be attributed to differences in allele frequencies of genetic markers. This can arise from inconsistent linkage disequilibrium phases between quantitative trait loci (QTL) and markers between subpopulations or breeds, or from different QTL segregating within groups. Both aspects have been used in the past to explain why across-breed genomic predictions based on 50 k genotypes have low accuracy [36–38].

As in the classical approach for modeling base groups, we assigned base animals to groups and calculated a matrix of genetic contributions **Q** using standard methodology. This matrix **Q** was then used to estimate average allele frequencies using mixed-model methodology, as described by Gengler et al. [21]. As mentioned in the Methods section, estimation of average allele frequencies in base groups is not essential for the proposed decomposition of **G**. However, it provides a convenient way to integrate new animals under practical conditions. Conceptually, it divides the genetic distance between any pair of animals into two parts, i.e. a distance that already exists in the base population and a distance that originates from the history of the breed as documented by the known pedigree. Moreover, estimating allele frequencies in base groups from subsets of genotypes may lead to similar problems as in standard applications of models that fit genetic groups, i.e., if the amount of data to estimate allele frequencies in base groups reliably is not sufficient, it can result in a loss of accuracy and introduction of bias [39]. Then, this tradeoff between defining all possible relevant base groups and estimability needs to be taken into account. A closer examination of the required size and properties for an optimal design of base groups is beyond the scope of this paper.

Group effects were not accounted for when deregressing MACE breeding values for BS/OB animals because (i) group effects or group contributions are usually not reported to Interbull by the participating countries; (ii) Interbull introduces its own group categorizations based on birth year of bull dams for MACE evaluation; and (iii) Interbull does not report group effects or group contributions back to the participating countries. Because of these limitations, we cannot exclude that our results for BS/OB animals may be influenced in one way or the other by the properties of MACE breeding values.

Since we tested different models only in a single forward prediction, the generalization of our results is not straightforward. However, from a practical point of view, the steps that we followed allowed us to better characterize the genetic composition of the validation groups. This in turn might help to decide if a standard GBLUP model is sufficient or whether a different model should be preferred. However, modeling genetic groups in any of the proposed ways is neither intended nor expected to improve the prediction for a standard animal with a pedigree that has many generations and that is sufficiently complete. Predictions for an animal with an incomplete pedigree or a limited number of genotyped ancestors should, however, benefit from the inclusion of group effects in one form or the other.

### Models

We compared three models, which treated effects of base groups as random (M0), as fixed (M2), or ignored them completely (M1). Model M1 consistently showed the lowest R^2^ values across both breeds and all traits. This was expected, since ignoring part of the genomic information should not result in increased predictive ability. However, it is interesting to note that the segregation term itself results in a relatively good prediction. Using M1, we observed differences in the decrease of the model R^2^ between traits, with the UD trait being the least influenced by **G**_A_^*^. We cannot exclude that there might be cases where omission of base groups will increase the R^2^ of predictions. However, the slopes of the regression of current DYD or deregressed proofs on DGV that we used as a test statistic here gave no indication that omitting **G**_A_^*^ without adjusting the genetic variance could lead to less inflated estimates. Recently, Makgahlela et al. [14] compared predictions using a genomic relationship matrix based on average allele frequencies across breeds with predictions using breed-specific allele frequencies in the Nordic Red dairy cattle population. This comparison is conceptually quite close to what we did in the comparison between the reduced model (M1) and the fixed model (M2**)**. The authors found a smaller predictive power and greater inflation of DGV when considering breed-specific allele frequencies. Since using breed-specific allele frequencies without modeling differences in allele frequencies in the base population is equivalent to our reduced model (M1), in this respect, their results are consistent with those presented here.

In terms of predictive power, M2 was better than M0 for all milk traits and one conformation trait for the BS/OB data (Table 5). With the FV data, we saw a clear advantage of M0 for all traits. In a preliminary study [40], we had reported that the OB and current BS populations were separated by a fairly large genetic distance. The validation BS/OB group that we used here included only very few OB animals. The observed genetic distance and the fact that this group of animals is small compared to the overall validation group might explain the small superiority of M2 observed for the BS/OB data. Genetic distances of similar magnitude were not detected in the FV population, for which M0 was clearly the best model. However, the German-Austrian cooperation for genetic evaluations in FV [22] recently fully opened the routine evaluations for the Czech population, which shows some differences in genetic composition compared to the current German-Austrian breeding population (Table 9). Additional investigations will be necessary to verify if M0 is still superior with an extended base population that will very likely be the result of this extended cooperation.

### Genetic contributions and base group effects

Analysis of the matrix of genetic contributions **Q** revealed some interesting features. For example, on the one hand, the analysis of average contributions of genetic groups to current animals revealed that US animals had a strong impact on the current BS population in Europe. On the other hand, a substantial contribution of the “old” European base group (*EU*_*b*_) to the *OB* validation group was found. Averages and standard deviations of contributions are also an indirect indicator for how accurate base allele frequencies and base group effects could be estimated from the current data. However, since information in **Q** naturally implies some degree of collinearity, this factor has to be taken into account also. Finally, differences in trait means between base groups can only be detected if there is enough variation in base group contributions within the training set (*Calib*). Such variation was observed for both breeds and was considerably smaller for the dominant groups of the validation set. This was expected since, in the last 20 years, much less migration has occurred in both populations, which probably resulted in less admixture in the more recent groups. Although this was not the primary focus of this investigation, it was interesting to note the extremely strong genetic contribution of American Brown Swiss animals to the current BS population. The validation group *OB* was clearly an exception in the sense that a small or even non-existing contribution of American Brown Swiss cattle defines what an OB animal is. In contrast, the strong contribution of the DE_b2_ group to the FV population seems to be an artifact of the completeness of the pedigree used, i.e. most of the pedigrees traced back to this base group.

For both breeds and for the traits analyzed here, it was possible to estimate significant differences between the means of base groups in most cases (Tables 6 and 7). Treating base groups as fixed or random resulted in similar patterns, although they were more pronounced in the case of fixed effects. The observed effects were quite consistent with our expectations and seem to be reasonable when considering the limits that were imposed on estimability and precision by the collinearity and dependencies in **Q** (**Q** has no full column rank). For example, the two Holstein base groups in the FV dataset had a clear advantage for protein yield, which is not surprising since Holstein bulls were introgressed for exactly that reason. In some cases, such as the advantage found for the DE_b_ group in BS, knowing that the base group definition for DE_b_ also comprised relatively young base animals was helpful, whereas assignment to American Brown Swiss was more linked to a specific period further back in the history of the breed.

Both the distribution of genetic contributions and precision of base group effects emphasize that when considering genetic grouping in genetic evaluation models, the question of estimability and relevance for the current population should always be included [39]. However, as already noted above, it is not reasonable to believe that the model used has a strong impact on predictive power if the animals used for validation show no differences in their genetic composition with respect to the base groups and if the majority of them have complete pedigrees of sufficient depth.

### Additional considerations

This investigation demonstrates that, in many cases, the genomic relationship matrix includes an important component of variation that has no corresponding counterpart in the conventional numerator relationship matrix. However, many practical applications of the estimation of GBV include a step for scaling the genomic relationship matrix to the numerator relationship matrix to set them on the same genetic base (see for example [41]). Based on our results, it seems more suitable to do this scaling based on matrix **G**_**S**_ only. This component of the **G**-matrix should be free of the effects of systematic differences in allele frequencies between base groups (represented in **G**_A_^*^), which might otherwise exacerbate the derivation of correct scaling factors. This issue was also raised by Makgahlela et al. [14] and might be of special importance for applications of one-step genomic evaluations [16, 17, 42, 43]. Furthermore, it suggests that estimating genetic parameters for genomic evaluations using **G**_**T**_ might be preferred over a simple transfer of the parameters estimated with the numerator relationship matrix.

Possible extensions of M0, for example with an individual λ for group effects or – in the most general form – using an identity matrix instead of **G**_**A**_, e.g. [39], as well as an individual λ for group effects were beyond the scope of this paper. In addition, these extensions would require the estimation of a variance component for groups, which would be difficult to do due to the typically small number of degrees of freedom for the variance between group means. Using **G**_**A**_ but assuming an individual λ for group effects is also somewhat questionable from a conceptual point of view, since it would be necessary to describe the covariance between and within subpopulations based on the same distance between allele frequencies but with different genetic variances.

## Conclusions

We showed that the proposed decomposition of the **G**-matrix is helpful to examine the relative importance of base group and segregation effects in a dataset. The commonly used genomic relationship matrix **G** is equivalent to our model M0, where base groups and segregation terms are considered as random effects with the same genetic variance. Although it is interesting to examine contributions of different founder populations from a scientific point of view, we also conclude that the standard model M0 is preferred in many cases, e.g. if base group effects are small or difficult to estimate, or if the current population is homogenous with balanced base group contributions. However, a fixed model (M2) might be preferred if base group effects are large (i.e. in the range of differences between breeds rather than between subpopulations) or if the genomic evaluation comprises two or more separated populations with only weak genetic links.

## Appendix 1

Proof that model 2 (fixed group effects model using **G**_S_ as covariance of individual genetic values) and a corresponding model using **G**_T_ as covariance of individual genetic values will lead to identical solutions for fixed and random effects.

As shown in Appendix 2, the standard model and model 0 are equivalent. Following that, BLUP solutions of a model using **G**_T_ as covariance of breeding values can be equivalently written as:

$$ \widehat{\mathbf{u}}={\mathbf{G}}_{\mathrm{S}}{\mathbf{V}}_{\mathrm{yy}}^{\hbox{-} \mathbf{1}}\tilde{\mathbf{y}}+\mathbf{Q}{\mathbf{G}}_{\mathrm{A}}\mathbf{Q}\mathbf{\hbox{'}}{\mathbf{V}}_{\mathrm{yy}}^{\hbox{-} \mathbf{1}}\tilde{\mathbf{y}}, $$

where **Q** is a matrix of genetic contributions of random groups to animals with observations as described in Methods and **ỹ** is the vector of observations corrected for the GLS-estimates of fixed effects. If the same matrix **Q** is used to model the fixed group effects, as it is generally done, this might be written as:

$$ \widehat{\mathbf{u}}={\mathbf{G}}_{\mathrm{S}}{\mathbf{V}}_{\mathrm{yy}}^{\hbox{-} \mathbf{1}}\left(\mathbf{y}\hbox{-} \mathbf{Q}\widehat{\mathbf{b}}\right)+\mathbf{Q}{\mathbf{G}}_{\mathrm{A}}\mathbf{Q}\hbox{'}{\mathbf{V}}_{\mathrm{yy}}^{\hbox{-} \mathbf{1}}\left(\mathbf{y}\hbox{-} \mathbf{Q}\widehat{\mathbf{b}}\right). $$

By omitting the global mean since it cannot be estimated simultaneously and by replacing $\widehat{\mathbf{b}}$ by its GLS-estimate, this can be further manipulated to give:

$$ \widehat{\mathbf{u}}={\mathbf{G}}_{\mathrm{S}}{\mathbf{V}}_{\mathrm{yy}}^{\hbox{-} \mathbf{1}}\left(\mathbf{y}\hbox{-} \mathbf{Q}\widehat{\mathbf{b}}\right)+\mathbf{Q}{\mathbf{G}}_{\mathrm{A}}\mathbf{Q}\hbox{'}{\mathbf{V}}_{\mathrm{yy}}^{\hbox{-} \mathbf{1}}\left(\mathbf{y}\hbox{-} \mathbf{Q}\widehat{\mathbf{b}}\right) $$

$$ ={\mathbf{G}}_{\mathrm{S}}{\mathbf{V}}_{\mathrm{yy}}^{\hbox{-} \mathbf{1}}\left(\mathbf{y}\hbox{-} \mathbf{Q}\widehat{\mathbf{b}}\right)+\mathbf{Q}{\mathbf{G}}_{\mathrm{A}}\mathbf{Q}\hbox{'}{\mathbf{V}}_{\mathrm{yy}}^{\hbox{-} \mathbf{1}}\mathbf{y}\hbox{-} \mathbf{Q}{\mathbf{G}}_{\mathrm{A}}\mathbf{Q}\hbox{'}{\mathbf{V}}_{\mathrm{yy}}^{\hbox{-} \mathbf{1}}\mathbf{Q}\widehat{\mathbf{b}} $$

$$ ={\mathbf{G}}_{\mathrm{S}}{\mathbf{V}}_{\mathrm{yy}}^{\hbox{-} \mathbf{1}}\left(\mathbf{y}\hbox{-} \mathbf{Q}\widehat{\mathbf{b}}\right)+\mathbf{Q}{\mathbf{G}}_{\mathrm{A}}\mathbf{Q}\hbox{'}{\mathbf{V}}_{\mathrm{yy}}^{\hbox{-} \mathbf{1}}\mathbf{y}\hbox{-} \mathbf{Q}{\mathbf{G}}_{\mathrm{A}}\mathbf{Q}\hbox{'}{\mathbf{V}}_{\mathrm{yy}}^{\hbox{-} \mathbf{1}}\mathbf{Q}\left(\mathbf{Q}\hbox{'}{\mathbf{V}}_{\mathrm{yy}}^{\hbox{-} \mathbf{1}}\mathbf{Q}\right){}^{-1}\mathbf{Q}\hbox{'}{\mathbf{V}}_{\mathrm{yy}}^{\hbox{-} \mathbf{1}}\mathbf{y} $$

$$ ={\mathbf{G}}_{\mathrm{S}}{\mathbf{V}}_{\mathrm{yy}}^{\hbox{-} \mathbf{1}}\left(\mathbf{y}\hbox{-} \mathbf{Q}\widehat{\mathbf{b}}\right)+\mathbf{Q}{\mathbf{G}}_{\mathrm{A}}\mathbf{Q}\hbox{'}{\mathbf{V}}_{\mathrm{yy}}^{\hbox{-} \mathbf{1}}\mathbf{y}\hbox{-} \mathbf{Q}{\mathbf{G}}_{\mathrm{A}}\mathbf{Q}\hbox{'}{\mathbf{V}}_{\mathrm{yy}}^{\hbox{-} \mathbf{1}}\mathbf{y} $$

$$ ={\mathbf{G}}_{\mathrm{S}}{\mathbf{V}}_{\mathrm{yy}}^{\hbox{-} \mathbf{1}}\left(\mathbf{y}\hbox{-} \mathbf{Q}\widehat{\mathbf{b}}\right), $$

and identical solutions for the random effect **u** are the consequence. It follows that the product of the design matrix **Q** and the contrast for the random group effect (represented by the second term above) is also zero, which is a necessary prerequisite for the resulting estimates, for the fixed genetic groups to be equal in both models also [44]. As a general consequence of the cited publication [44], any extension of **V** in the GLS-estimate of **b** of the form:

$$ {\mathbf{V}}^{*}=\mathbf{V}+\mathbf{X}\mathbf{U}\mathbf{X}\hbox{'}, $$

for an arbitrary matrix **U**, where **X** is the same design matrix used to estimate the fixed effect itself, results in GLS-estimates for the fixed effects that are identical to those using **V** alone [44].

## Appendix 2

Proof that the standard model is equivalent to the random group model M0.

Let the standard model be:

$$ \mathbf{y}=\mathbf{X}\mathbf{b}+\mathbf{Z}\mathbf{u}+\mathbf{e}, $$

where **y** is a vector of observations, **b** is a vector of fixed effects, **u** is a vector of random breeding values, **e** is a vector of residuals and **X** and **Z** are known design matrices. For simplification of the presentation **Z** is assumed to be an identity matrix and is omitted. Furthermore, **y** ~ N(**Xb, V**_yy_), **u** ~ N(**0**,**V**_uu_) and **e** ~ N(**0**,**V**_e_) where:

$$ {\mathbf{V}}_{\mathrm{e}}=\mathbf{I}\times {\upsigma}_{\mathrm{e}}^2=\mathbf{R}, $$

$$ {\mathbf{V}}_{\mathrm{u}\mathrm{u}}={\tilde{\mathbf{G}}}_{\mathrm{T}} \times {\upsigma}_{\mathrm{u}}^2={\mathbf{G}}_{\mathrm{T}}, $$

and

$$ {\mathbf{V}}_{\mathrm{yy}}={\mathbf{G}}_{\mathrm{T}}+\mathbf{R}. $$

Assume a decomposition of the coefficient matrix ${\tilde{\mathbf{G}}}_{\mathrm{T}}=\left({\tilde{\mathbf{G}}}_{\mathrm{S}}+{\tilde{\mathbf{G}}}_{\mathrm{A}}\right)\times {\upsigma}_{\mathrm{u}}^2={\mathbf{G}}_{\mathrm{S}}+{\mathbf{G}}_{\mathrm{A}}^{*}$ where **G**_A_^*^ can be expressed as the product of a matrix of fixed regression coefficients **Q** and a matrix **GA**, that describes the covariance of random slopes, so **G**_A_^*^ = **QG**_**A**_**Q** '**.** The BLUP estimates for random breeding values are:

$$ \widehat{\mathbf{u}} = {\mathbf{G}}_{\mathrm{T}}{\mathbf{V}}_{\mathrm{yy}}^{\hbox{-} \mathbf{1}}\left(\mathbf{y}\hbox{-} \mathbf{X}\widehat{\mathbf{b}}\right) = {\mathbf{G}}_{\mathrm{T}}{\mathrm{V}}_{\mathrm{yy}}^{\hbox{-} \mathbf{1}}\tilde{\mathbf{y}}, $$

with $\widehat{\mathbf{b}}$ being the generalized least squares estimates of **b**. It follows that:

$$ {\mathbf{V}}_{\mathrm{yy}}={\mathbf{G}}_{\mathrm{T}}+\mathbf{R} $$

$$ ={\mathbf{G}}_{\mathrm{S}}+{\mathbf{G}}_{\mathrm{A}}^{*}+\mathbf{R} $$

$$ ={\mathbf{G}}_{\mathrm{S}}+\mathbf{Q}{\mathbf{G}}_{\mathrm{A}}\mathbf{Q}\hbox{'}+\mathbf{R}, $$

and

$$ \widehat{\mathbf{u}} = {\mathbf{G}}_{\mathrm{T}}{\mathbf{V}}_{\mathrm{yy}}^{\hbox{-} \mathbf{1}}\tilde{\mathbf{y}} $$

$$ =\left({\mathbf{G}}_{\mathrm{S}}+{\mathbf{G}}_{\mathrm{A}}^{*}\right){\mathbf{V}}_{\mathrm{yy}}^{\hbox{-} \mathbf{1}}\tilde{\mathbf{y}} $$

$$ =\left({\mathbf{G}}_{\mathrm{S}}+\mathbf{Q}{\mathbf{G}}_{\mathrm{A}}\mathbf{Q}\hbox{'}\right){\mathbf{V}}_{\mathrm{yy}}^{\hbox{-} \mathbf{1}}\tilde{\mathbf{y}} $$

$$ ={\mathbf{G}}_{\mathrm{S}}{\mathbf{V}}_{\mathrm{yy}}^{\hbox{-} \mathbf{1}}\tilde{\mathbf{y}}+\mathbf{Q}{\mathbf{G}}_{\mathrm{A}}\mathbf{Q}\hbox{'}{\mathbf{V}}_{\mathrm{yy}}^{\hbox{-} \mathbf{1}}\tilde{\mathbf{y}}. $$

Let the random group model be:

$$ \mathbf{y} = \mathbf{X}\mathbf{b}+\mathbf{Z}\mathbf{u} + \mathbf{Q}\mathbf{g}+\mathbf{e}, $$

where **y** is a vector of observations, **b** is a vector of fixed effects, **u** is a vector of random genetic values, **g** is a vector of random group effects, **e** is a vector of residuals and **X** and **Z** are known design matrices. For simplification of the expressions, **Z** is assumed to be an identity matrix and is omitted. **Q** is a matrix of genetic contributions of random groups to animals with observations as described in Methods. Furthermore, **y** ~ N(**Xb, V**_yy_), **u** ~ N(**0**,**V**_uu_), **g** ~ N(**0**,**V**_gg_) and **e** ~ N(**0**,**V**_e_) where:

$$ {\mathbf{V}}_{\mathrm{e}}=\mathbf{I}\times {\upsigma}_{\mathrm{e}}^2=\mathbf{R}, $$

$$ {\mathbf{V}}_{\mathrm{u}\mathrm{u}}={\tilde{\mathbf{G}}}_{\mathrm{S}}\times {\upsigma}_{\mathrm{u}}^2={\mathbf{G}}_{\mathrm{S}}, $$

$$ {\mathbf{V}}_{\mathrm{gg}}={\tilde{\mathbf{G}}}_{\mathrm{A}}\times {\upsigma}_{\mathrm{u}}^2={\mathbf{G}}_{\mathrm{A}}, $$

$$ {\mathbf{V}}_{\mathrm{yy}} = {\mathbf{G}}_{\mathrm{S}}+\mathbf{Q}{\mathbf{G}}_{\mathbf{A}}\mathbf{Q}\hbox{'} + \mathbf{R}, $$

$$ ={\mathbf{G}}_{\mathrm{S}}+{\mathbf{G}}_{\mathrm{A}}^{*}+\mathbf{R}. $$

This is identical to the phenotypic variance assumed by the standard model if the same **Q** is used.

The BLUP solutions for random animal and group effects are:

$$ \widehat{\mathbf{u}} = {\mathbf{G}}_{\mathrm{S}}{\mathbf{V}}_{\mathrm{yy}}^{\hbox{-} \mathbf{1}}\left(\mathbf{y}\hbox{-} \mathbf{X}\widehat{\mathbf{b}}\right)={\mathbf{G}}_{\mathrm{S}}{\mathbf{V}}_{\mathrm{yy}}^{\hbox{-} \mathbf{1}}\tilde{\mathbf{y}}, $$

and

$$ \widehat{\mathbf{g}}={\mathbf{G}}_{\mathrm{A}}\mathbf{Q}\hbox{'}{\mathbf{V}}_{\mathrm{yy}}^{\hbox{-} \mathbf{1}}\left(\mathbf{y}\hbox{-} \mathbf{X}\widehat{\mathbf{b}}\right) = {\mathbf{G}}_{\mathrm{A}}\mathbf{Q}\hbox{'}{\mathbf{V}}_{\mathrm{yy}}^{\hbox{-} \mathbf{1}\ }\tilde{\mathbf{y}}. $$

Let the full estimate for the breeding value (the ranking criterion) be:

$$ ={\mathbf{G}}_{\mathrm{S}}{\mathbf{V}}_{\mathrm{y}\mathrm{y}}^{\hbox{-} \mathbf{1}}\tilde{\mathbf{y}} + \mathbf{Q}{\mathbf{G}}_{\mathrm{A}}\mathbf{Q}\hbox{'}{\mathbf{V}}_{\mathrm{y}\mathrm{y}}^{\hbox{-} \mathbf{1}\ }\tilde{\mathrm{y}}, $$

this is identical to the breeding value solution of **û** of the standard model if **Q** is identical in both models.
